# The role of angiotensin receptor blocker (losartan) on decreasing fibrotic process of corpora cavernosa in priapism model of wistar rats

**DOI:** 10.12688/f1000research.123040.3

**Published:** 2024-02-06

**Authors:** Safendra Siregar, Rulianov Rulianov, Rangga Adhazi Ksatriapraja, Dicky Stefanus

**Affiliations:** 1 Department of Urology, Universitas Padjadjaran, Bandung, 40161, Indonesia

**Keywords:** Angiotensin II receptor blocker, Losartan, Collagen, Transforming Growth Factor, Priapism, collagen type I, fibrosis, renin-angiotensin II system

## Abstract

**Background:**

Priapism induces regulation of Transforming Growth Factor-β1 (TGF-β1) expression and collagen-type-1 deposition. This will replace the normal corpora cavernosa with fibrotic tissue which eventually resulted in erectile dysfunction. It is also known that the fibrosis process of corpora cavernosa is related to Renin-Angiotensin II System (RAS). Angiotensin II receptor blockers (ARB), especially losartan, inhibit the inflammation process and fibrotic tissue formation. This study evaluated the effect of losartan in reducing fibrosis in priapism by evaluating TGF-β1 and collagen-type-1 in cavernous tissue and determined the effect of losartan in preventing fibrosis in priapism model of Wistar rats assessed by the metavir score.

**Methods:**

A total of eighteen male Wistar rats mean were divided into five groups. For the priapism models, we applied negative pressure on the penis to make an artificial erection to mimic the priapism process. The control groups were observed and the treatment groups were orally given losartan 15 mg/kg/day. *Corpora cavernosa* was harvested for TGF-β1 and collagen-type-1 measurement using an enzyme-linked immunosorbent assay (ELISA). The fibrotic tissue of each rat was then collected and assessed histopathologically with the metavir scoring system.

**Results:**

Penile TGF-β1 concentration in the losartan-treated group was not significantly different on day 10 and day 28 of observation (p10=0,30; p28=0,17). Meanwhile, collagen-type-1 concentration was significantly lower compared to control group (p10=0,002; p28=0,01). There was a significant difference in metavir scores in rats that received losartan and those who did not (p<0,05).

**Conclusion:**

Losartan could suppress the fibrosis process in the priapism model. It could decrease the collagen type 1 deposition during *corpora cavernosa* tissue regeneration. Based on the metavir score, the group receiving losartan therapy was better than the control group.

## Introduction

Priapism is defined as prolonged erection more than four hours after sexual stimulation with orgasm or without any sexual stimulation. ^1^^–^^4^ The prevalence of this urology emergency condition was 5,34 cases per 100.000 males per year. ^2^ Based on its pathophysiology, priapism can be classified into the high and low flow. ^1^^,^^2^ Low flow priapism correlated with venous occlusion which increases internal corpus cavernous pressure and prevents arterial blood flow. ^1^^,^^5^^,^^6^ Intervention more than 48 hours prevent structural recovery which leads to erectile dysfunction. ^1^^,^^3^^,^^5^

During hypoxia condition, in the first 12 hours, histological examination showed interstitial edema in the corporal specimen. This condition is followed by endothelial sinusoid injury, inflammation cell infiltration to basal membrane, and platelet aggregation after 24 hours. In 48 hours, thrombus can be found in sinusoids with smooth muscle necrosis and fibroblast proliferation. ^3^^–^^5^ Hypoxia-induced growth factors increase during hypoxia environment such as Transforming Growth Factor 1 (TGF beta 1). Overexpression of TGF beta 1 leads to tissue injury and scar formation. ^1^^,^^3^ TGF beta 1 recruits fibroblast to form molecular matrices such as fibronectin and collagen to build extracellular matrix and increase protease inhibitor production which inhibits protease degrades the extracellular matrix. ^7^

A study by Costa *et al.* showed that corporal cavernous tissue in the adult penis with a history of priapism had a decreased smooth muscle component with increased type 1 collagen compared to them without priapism history. Various therapy has been carried out to manage the fibrotic condition in the corpora cavernosa, with the hope of treating erectile dysfunction conditions which include medical and surgical therapy. Phosphodiesterase-5 inhibitor (PDE5-I) and pentoxiphylline were an example of medicine while penile implants are one of the surgical options. ^4^ Unfortunately, both medical and surgery focuses more on restoring erectile function than preventing the formation of fibrotic tissue which causes Erectile Dysfunction (ED).

The hypoxic state in the *corpora cavernosa* can be a factor that initiates fibrosis in the corpora cavernosa via the angiotensin II-Smad pathway which is initiated by activation of the renin-angiotensin II system (RAS). Angiotensin II will bind to the AT1 receptor and cause an increase in TGF-β1 and activate the smad pathway. AngII through the AT1 receptor also increases tissue inhibitor of matrix metalloproteinase-1 (TIMP-1) to inhibit matrix metalloproteinase (MMP) which functions in the degradation (turnover) of ECM proteins in the remodeling process. Changes in the histological picture of the corpora cavernosa in the form of a decrease in the number of smooth muscle cells replaced by collagen caused a decrease in the ratio of smooth muscle and collagen and an increase in fibroblasts which could be assessed by Masson’s Trichrome staining with the metavir scoring system.

Currently, there are several ways of assessing fibrosis in the corpora cavernosa such as the ratio of smooth muscle to collagen, but none of them have assessed the lumen of the cavernosa which is the most important part of the penile erection process, so the metavir scoring system is considered the best for assessing the purpose of this study. ^8^

Angiotensin II receptor blockers (ARBs) are antihypertensive drugs widely used to inhibit the inflammatory process and the formation of fibrotic tissue. The types of ARBs commonly used are losartan, candesartan, eprosartan mesylate, irbesartan, telmisartan, and valsartan. ^6^^,^^7^^,^^9^ Several studies have demonstrated the ability of ARBs as tissue antifibrosis by inhibiting angiotensin II receptor type 1, thereby reducing the formation of TGF-β1. This can be seen in the study by Yamamoto *et al*. regarding the effect of ACE-I and ARB in reducing fibrosis in the heart ventricles that occurs due to hypertension. ^9^ Losartan is one of the first-generation ARB drugs that have good anti-fibrotic properties. The administration of losartan in various studies has been shown to reduce the incidence of heart, liver, and kidney fibrosis. ^10^^–^^12^

The anti-fibrotic ability of losartan gives hope to overcome the fibrotic process that occurs in various pathological conditions in the human body, including the penis.

This study aimed to evaluate the anti-fibrotic effect of losartan in reducing TGF-β1 and type 1 Collagen in the priapism model and to determine the effect of losartan administration on the process of corpora cavernous fibrosis in the priapism model of Wistar rats assessed by the metavir score.

## Methods

This was an experimental study. Sample size calculation was done using Federer formula (T-1) (N-1) ≥ 15. Total of eighteen male Wistar rats with an average weight of 328 grams were divided into five groups which are:

• Group I (without ARB therapy): Priapism model rats, sacrificed on day ten
• Group II (without ARB therapy): Priapism model rats, sacrificed on day 28
• Group III (ARB therapy): Priapism model rats given orally losartan, sacrificed on day ten
• Group IV (ARB therapy): Priapism model rats given orally losartan, sacrificed on day 28
• Group V (healthy control): Untreated mice, sacrificed on day 28

The inclusion criteria were as follows: male Wistar rat, rat bodyweight 300–350 grams, has no congenital or acquired disease, and is active. Other confounders other than our inclusion criteria were not controlled. For the priapism models, we applied negative pressure on the penis to make an artificial erection and hold the base of the penile shaft using a rubber band to mimic the priapism process. After 12 hours, the rubber band was released. The control groups were then observed and the treatment groups were given losartan 15 mg/kg body weight dissolved in drinking water oral route with nasogastric tube per day. Half of the groups were euthanized on day ten and the rest on day 28. Following euthanasia, *corpora cavernosa* was harvested for TGF-β1 and collagen type 1 measurement using Enzyme-Linked Immunosorbent Assay (ELISA).

The procedure for making a low flow type of priapism model was carried out on 18 adult male Wistar rats included in the study. The cages were cleaned daily and the temperature was maintained between 26–29 degrees Celsius. The temperature was measured using a thermometer and monitored every morning. Every morning the rat cage was left exposed to the sun from 8 to 10 am, and the cage was cleaned at the same time. After being given the same treatment and resting for a week, the samples were divided into five groups. The manufacture of the priapism model was carried out in collaboration with experienced and certified laboratory personnel using the method that has been carried out and has been published by Sanli, *et al.* ^18^

This method was performed on 16 male Wistar rats group I to IV which were anesthetized intraperitoneally by injection of ketamine 75 mg/kg body weight. Meanwhile, two rats in group V were not given any treatment. Furthermore, we performed negative pressure on the penis by placing the tip of the 50 mL catheter tip around the penis and slowly pulling the syringe until the penis is fully erect. The negative pressure exerted was equal to the pull at 20 ccs. Then ligation at the base of the penis by tying a piece of 16 Fr rubber catheter that had been cut 2 mm long. This was done so that hypoxia occurs in the *corpora cavernosa* of the rat penis resembling priapism.

The rubber band was then removed after 12 hours to restore blood flow to the corpora cavernosa penis of the rat. Following the removal of the rubber band, groups III and IV were given ARB losartan 15 mg/kg body weight/day orally with the help of NGT number 6 Fr for 10 days and 28 days. The mice in groups I and III were sacrificed after ten days and group II, IV, and V mice were sacrificed after 28 days. Rats were sacrificed by euthanasia injection of ketamine 300 mg/kg body weight intraperitoneally. Surgical removal of the corpora cavernosa was performed in each group. Rats were then exterminated using an incinerator.

### TGF-β1 and type 1 collagen assessment

The levels of TGF-β1 and type 1 collagen in the corpora cavernosa tissue samples were measured using the ELISA technique. The corpora cavernosa from the penile tissue was separated from other structures such as the urethra, corpora spongiosa, and tunica albuginea. Type 1 collagen in the tissue would stick to the plate which had been coated with type 1 collagen antibody. Before processing, the tissue was rinsed using Phosphate Buffer Saline (PBS) solution (pH 7.4) to remove excess blood and tissue. The tissue was then smoothed and homogenized in PBS solution (pH 7.4) with a glass homogenizer on ice. The tissue could be thawed at 2-8 **°**C or frozen at -20 **°**C if it was to be stored beforehand. Before the procedure, the sample tissue was allowed to reach room temperature and then centrifuged at 3000 RPM for 20 minutes.

### Metavir score assessment

The corpora cavernosa tissue samples were fixed in 10% formaldehyde solution. The sample is then processed and made into paraffin blocks. Paraffin-embedded tissue was sliced into 5-m sections and placed on an object-glass, followed by deparaffinization and rehydration. Hydrogen peroxide was administered for 10 minutes to eliminate endogenous peroxidase activity. The sample was then rinsed using PBS-Triton X 100 (Tx). Hematoxylin-eosin (HE) staining was performed to assess structural changes in the corpora cavernosa. To determine smooth muscle and collagen, cross-sections were stained with Masson’s trichrome (MT) using the MT staining kit. Areas of smooth muscle (red stain) and collagen (blue-green stain) were evaluated in 200× magnification images of tissue using a microscope. Corpora cavernous fibrosis was assessed after Masson’s trichrome staining with the metavir scoring system. Score 0 indicating absence of fibrosis; score 1 indicated mild fibrosis causes narrowing at lumens less than 25% of caverns; score 2 indicated moderate fibrosis causes narrowing at lumens about 25-50% of caverns; score 3 indicated moderate fibrosis causes narrowing at lumens more than about 50% of caverns; score 4 indicated severe fibrosis causes narrowing at lumens less than 75-100% of caverns; score 5 = severe fibrosis causes narrowing at all of caverns. ^14^ The observer was blinded during the examination of metavir score assessment.

### Statistical analysis

The data were processed using SPSS version 20.0 with Windows as the operating system. The normality test of the data was carried out with the Shapiro-Wilk test. Because the number of samples was less than 50. The p-value that was considered significant is 0.05. Parametric statistical test to assess the comparison of type I collagen and TGF-β1 between groups receiving losartan therapy with controls at each observation time using the unpaired T-test if the data was normally distributed or the Mann-Whitney test if the data were not normally distributed. Non-parametric statistical tests were used to assess meta var scores using the Kruskal-Wallis statistical test with posthoc analysis using the Mann-Whitney U test. This study has been approved by the Health Research Ethics of Universitas Padjadjaran Bandung with ethical protocol number: 329/UN6.KEP/EC/2021.

## Results

This study involved 18 male Wistar rats ( *Rattus norvegicus*) with an average weight of 328.1 grams at the beginning of the study. The samples were divided into five groups, namely four rats in group I (a model of priapism without therapy was sacrificed on day ten), four rats in group II (a model of priapism without therapy which was sacrificed on day 28), four rats in group III (a model of priapism with the ARB losartan therapy sacrificed on day ten), four mice in group IV (a model of priapism with the ARB losartan therapy sacrificed on day 28), and two mice in group V (healthy controls). All lived until the end of the study and none of the mice’s penises was necrotic. Each group was selected randomly and there was no significant difference in body weight in all rats (p-value on day 0 = 0.32; p-value on day 10 = 0.84; p-value on day 20 = 0.82; and p-value of day 20 = 0.82; to 28 = 0.54); p < 0.05).

### TGF-β1 concentration

The average concentration of TGF-β1 in the control group and the losartan ARB therapy group which were examined using the ELISA method on day 10 and day 28 can be seen in Table 1. The concentration of TGF-1 in the corpora cavernosa rats in the control group and the losartan ARB therapy group appeared to increase compared to the healthy control group on both the 10th and 28th-day observations. In the control group, the TGF-β1 concentration increased on day 28 (214.07 ± 41.85 ng/L) compared to day 10 (190.50 ± 10.72 ng/L). Meanwhile, in the losartan ARB therapy group, TGF-β1 concentrations appeared to decrease on day 28 (172.79 ± 29.77 ng/L) compared to day 10 (178.79 ± 17.88 ng/L).

**Table 1.** Concentration of TGF-β1 in Rat Corpora Cavernosa.

Observation time	TGF-β1 concentration (ng/L)		
	Healthy control group	Control group	Losartan ARB therapy group
**10th-day**			
Mean ± SD	157.68 ± 4.59	190.50 ± 10.72	178.79 ± 17.88
Range	154.43 – 160.93	181.25 – 205.98	164.25 – 202.13
**28th-day**			
Mean ± SD	157.68 ± 4.59	214.07 ± 41.85	172.79 ± 29.77
Range	154.43 – 160.93	170.66 – 260.10	143.41 – 207.65

**Figure 1.**
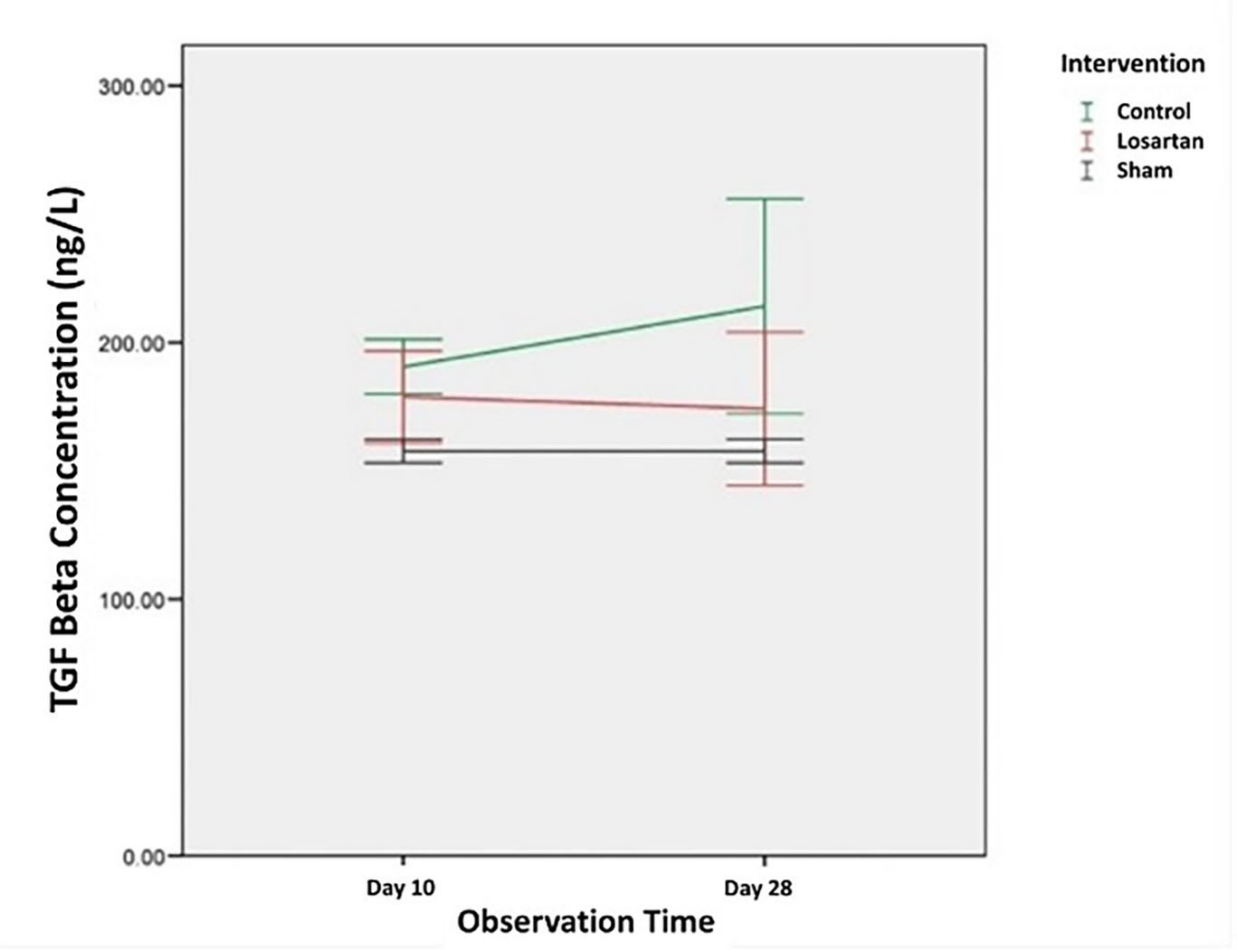
The concentration of TGF-β1 in Rat Corpora Cavernosa.

Changes in the concentration of TGF-β1 in all groups can be seen in Figure 1. From the graph, we can see that the concentration of TGF-β1 in the group modeled for priapism (the control group and the losartan ARB therapy group) was higher than the healthy control group. In the control group, the concentration of TGF-β1 tends to increase, while in the ARB losartan group it tends to decrease. If we compare the groups using the priapism model, it can be seen that the concentration of TGF-β1 in the group receiving the losartan ARB therapy is lower than in the control group.

### Type 1 collagen concentration

Table 2 shows the average value of type I collagen concentration in the control group and the losartan ARB therapy group on day ten and day 28. The concentration of type I collagen in the control group and the losartan ARB therapy group appeared higher than the healthy control group on both days of observation, the 10th and 28th days. In the control group, the concentration of type 1 collagen increased on day 28 (7.06 ± 0.44 ng/mL) compared to day 10 (6.81 ± 0.41 ng/mL). Likewise in the losartan ARB therapy group, the concentration of type 1 collagen also appeared to increase on day 28 (5.57 ± 1.18 ng/mL) compared to day 10 (5.46 ± 0.28 ng/mL).

**Table 2.** Type I collagen concentration in Corpora Cavernosa Rats.

Observation time	Type I collagen concentration (ng/mL)		
	Healthy control group	Control group	Losartan ARB therapy group
**10th-day**			
Mean ± SD	5.14 ± 0.40	6.81 ± 0.41	5.46 ±0.28
Range	4.86 – 5.43	6.25 – 7.26	5.30 – 5.90
**28th-day**			
Mean ± SD	5.14 ± 0.40	7.06 ± 0.44	5.57 ± 1.18
Range	4.86 – 5.43	6.52-7.50	4.19 – 6.89

**Figure 2.**
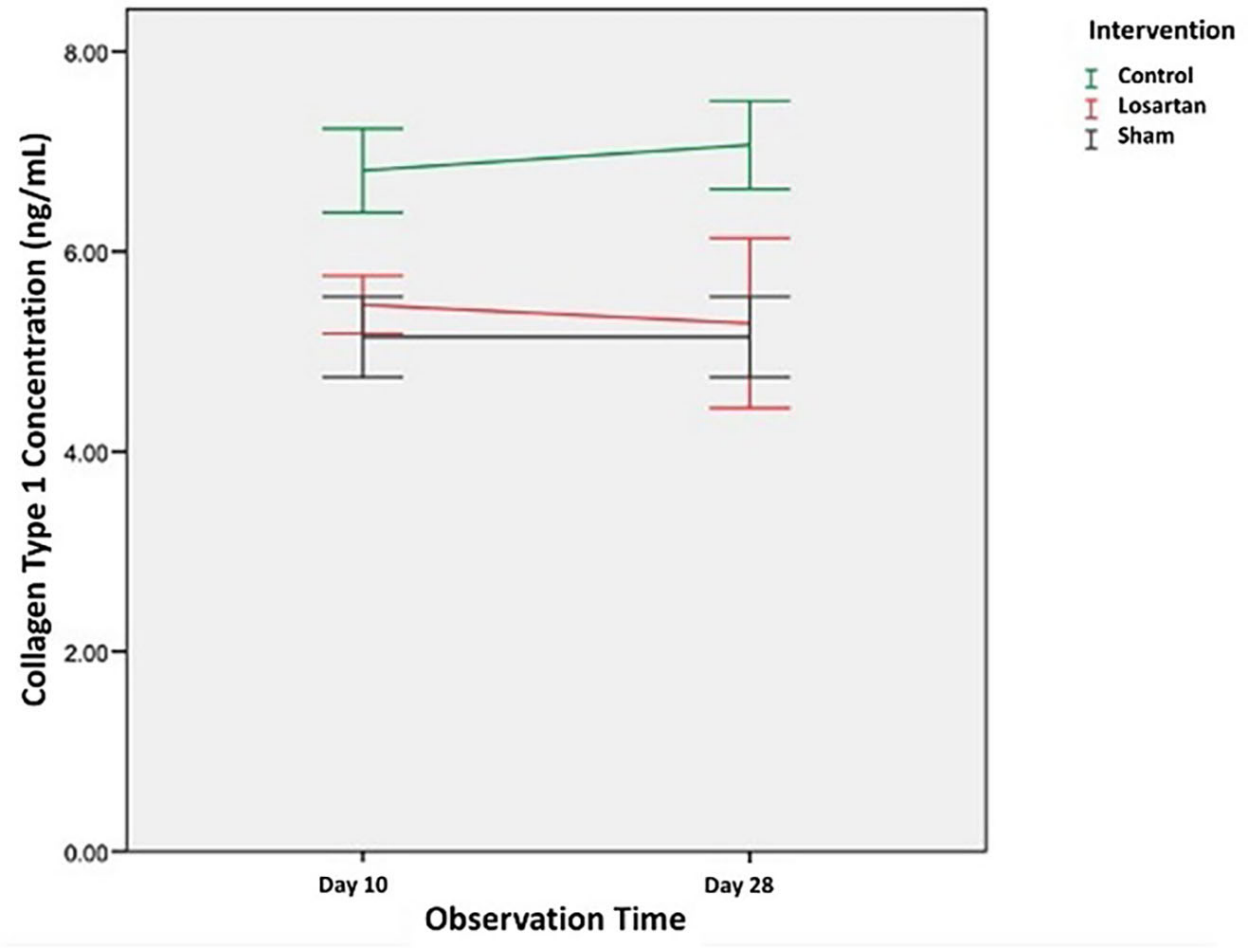
The concentration of Type I collagen in Rat Corpora Cavernosa.

In Figure 2 we can see changes in the concentration of type 1 collagen in all groups. From the graph, it can be seen that the concentration of type 1 collagen in the group modeled for priapism (control group and the losartan ARB therapy group) was higher than the healthy control group. In the group modeled for priapism, the concentration of type 1 collagen was equally likely to increase, but in the ARB losartan group, it was lower than in the control group.

### Comparison of TGF-β1 concentrations in the two groups based on observation time

Before the comparative analysis, a normality test was performed using the Shapiro-Wilk test and all data were normally distributed except for collagen levels in the losartan ARB therapy group on day ten, which was 0.010 (p < 0.05). However, due to the small number of samples, all of them were considered normally distributed.

Analytical comparative study between TGF-β1 concentration in both groups based on observation time using unpaired T-Test (p < 0.05). From Table 3, although not statistically significant, the difference in the concentration of TGF-β1 on day 10 in the losartan ARB group (178.9 ng/L) compared to the control group (190.50 ng/L) (p = 0.30, mean difference 11.71 ng/L, 95% Confidence Interval). At the time of observation on day 28, there was a difference in the concentration of type 1 collagen in the losartan ARB group (172.79 ng/L) compared to the control group (214.07 ng/L) (p = 0.17, a mean difference of 39.90 ng/L, 95% Confidence Interval).

**Table 3.** Mean concentration of TGF-β1 concentrations on day 10 and day 28 in the losartan ARB control and therapy group.

Observation time	TGF-β1 (ng/L) concentration		Mean difference (95% Confidence interval)	P value
	Control group (n=4)	ARB losartan therapy group (n=4)		
10th-day	190.50	178.79	11.71 (-13.80 – 37.22)	0.30 ^a^
28th-day	214.07	172.79	39.90 (-22.93 – 102.75)	0.17 ^a^

^a^ Independent T-Test.

### Comparison of type 1 collagen concentrations in the two groups based on observation time

Statistical analysis of the comparison of the concentration of type 1 collagen in the two groups based on the time of observation using the unpaired T-test (p < 0.05). From Table 4, there was a statistically significant difference in the concentration of type 1 collagen on day 10 in the losartan ARB group (5.46 ng/mL) compared to the control group (6.81 ng/mL) (p = 0.002, mean difference 1.34 ng/mL, 95% Confidence Interval). At the time of observation on day 28, there was also a statistically significant difference in the concentration of type 1 collagen in the losartan ARB group (5.57 ng/mL) compared to the control group (7.06 ng/mL) (p = 0.01, a mean difference of 1.78 ng/mL, 95% Confidence Interval).

**Table 4.** Mean concentration of collagen type 1 on day 10 and day 28 in the control group and losartan ARB therapy.

Observation time	Type 1 collagen concentration (ng/mL)		Mean difference (95% Confidence interval)	P value
	Control group (n = 4)	Losartan ARB therapy group (n = 4)		
10th-day	6.81	5.46	1.34 (0.71 – 1.96)	0.002 ^a^^*^
28th-day	7.06	5.57	1.78 (0.60 – 2.95)	0.01 ^a^^*^

^a^ Independent T-Test.

* Statistically significant.

### Metavir score

**Figure 3.**
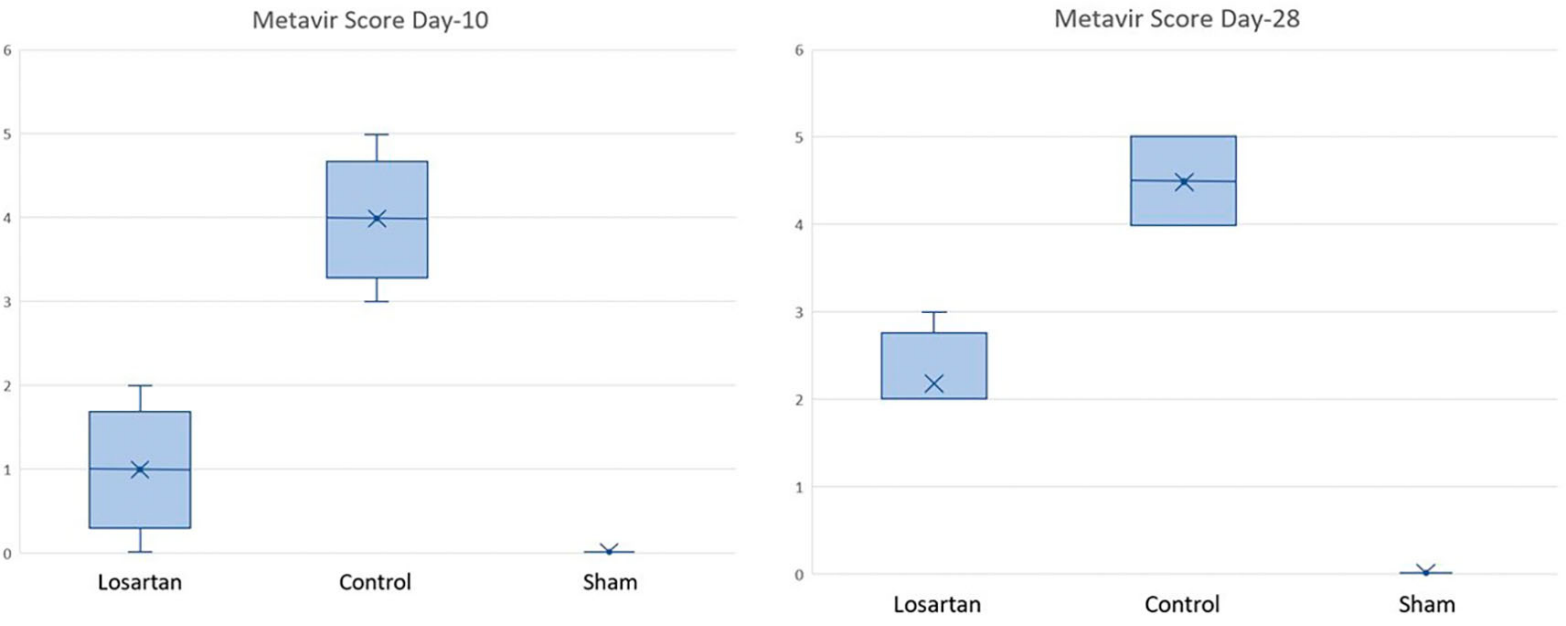
Graph of Metavir score in rats on days 10 and 28.

Table 5 shows the median metavir scores in the control and treatment groups, both of which were significantly higher at week two than at week four. There was a difference between the group receiving losartan compared to the control group. Changes in the score can be seen in Figure 3, which shows that the increase in metavir scores in the group receiving losartan therapy is lower than in the group that does not.

**Table 5.** Metavir score value.

Observation	Losartan group	Control group	Sham group	P value
Day-10				0.038 ^*^
Total (n)	4	4	1	
Median (range)	1 (0 – 2)	4 (3 – 5)	0	
Day-28				0.030 ^*^
Total (n)	4	4	1	
Median (range)	2 (2 – 3)	4.5 (4 – 5)	0	

* Kruskal-Wallis statistical test.

**Figure 4.**
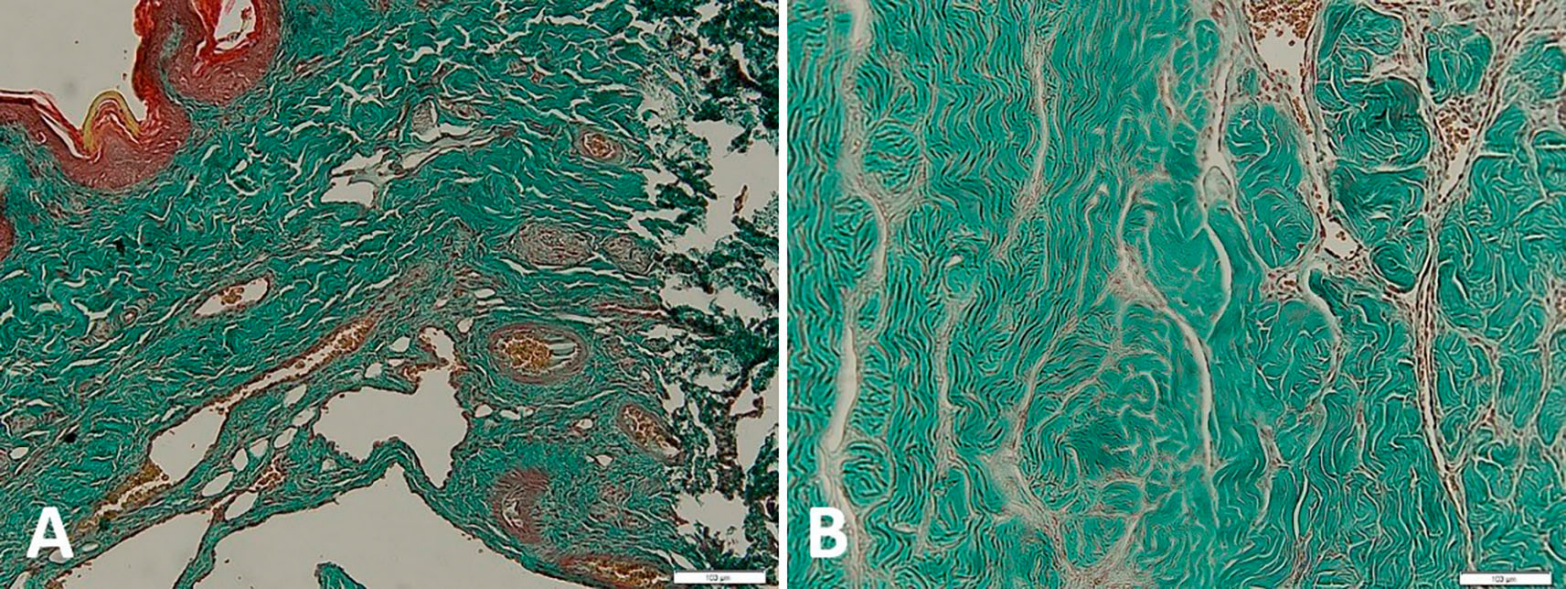
Histopathological appearance of the corpora cavernosa tissue with Masson trichrome staining: A with losartan therapy, B without losartan therapy shows the significant difference (red: muscle tissue, green: collagen).

Changes in metavir scores in the losartan therapy group and the control group are shown in Figure 3. Based on the graph, it can be seen that the metavir scores on day ten and day 28 looked different in the group given losartan and not. On day ten, the losartan group appeared to have lower metavir scores than the non-losartan group and closer to the Metavir score in the healthy group. Figure 4 shows the qualitative difference between the corpora cavernous tissue is given losartan and not in the case of priapism. In the *corpora cavernosa* treated with Losartan, the metavir score was lower.

Based on Table 6, it appears that there is a significant difference between the losartan therapy group and the control group (p = 0.019) on day ten and day 28. Based on Table 5 and Figure 3, there is a difference in metavir scores in the two groups, but the group receiving losartan therapy did better than the group that did not. On day 28, there was a significant difference in metavir scores in mice that received losartan and those that did not. It can be concluded that the administration of losartan can prevent the process of fibrosis in the *corpora cavernosa* of rats compared to controls that were assessed histopathologically with metavir scores.

**Table 6.** Metavir score post hoc analysis.

Day	Group		P value ^*^
10	Control	Sham	0.014
		Losartan therapy	0.019
	ARB therapy	Sham	0.264
		Control	0.019
28	Control	Sham	0.013
		Losartan therapy	0.017
	ARB therapy	Sham	0.049
		Control	0.017

* Post Hoc Mann-Whitney U test.

## Discussion

### TGF-β1 and type 1 collagen

In low-flow priapism, prolonged hypoxia occurs, causing histopathological changes of endothelial damage, exposure of the basement membrane, platelet aggregation, and changes in smooth muscle cells to fibroblasts. This mechanism caused the process of fibrosis in the corpora cavernosa tissue. ^15^^–^^17^ The process of tissue fibrosis can occur due to chronic diseases such as chronic disorders, heart failure, liver cirrhosis. Molecularly, this occurs because tissue hypoxia will increase the expression of angiotensin II which stimulates the AT1 receptor causing the synthesis of TGF-β1. TGF-β1 will stimulate fibroblasts to turn into myofibroblasts to produce type I collagen so that type 1 collagen deposits occur in the extracellular matrix and form fibrotic tissue. ^1^^,^^13^^,^^18^^,^^19^

This research was conducted in vivo on male Wistar rats. The priapism penis model is made by applying negative pressure to the penis by placing the tip of a 50 mL catheter tip around the penis and gently pulling the syringe until the penis is fully erect. The penis is then ligated by attaching a 16 Fr rubber catheter to create a priapism-like condition and then removed after 12 hours. According to research by Cleutjens J *et al.*, evaluation of ARB administration in liver fibrosis model mice on days seven, 14, and 28 showed that fibrosis improvement occurred at week two (day 14) and week four (day 28). ^20^ In general there are three phases in the occurrence of wound healing, namely: the hemostasis phase which lasts from the first day to the 5th day, the proliferative phase from day five to 12, and the remodeling phase which lasts for months. Fibroblast and collagen recruitment was assessed in this study. Therefore, sampling was carried out on day ten during the proliferative phase and day 28 during the remodeling phase.

In this study, there was an increase in TGF-β1 and type 1 collagen in the groups modeled for priapism (control group and the losartan ARB therapy group). Then we can also note that the increase in the concentration of TGF-β1 in the group receiving losartan therapy was lower than the control group on both the 10th and 28th-day observations. In addition, the concentration tends to decrease on the 28th day of observation compared to the 10th day.

This decrease in the concentration of TGF-β1 is in line with research by Garg *et al.* that can be helped by several anti-fibrotic agents, one of which is losartan. In this study, giving losartan 10 mg/kg/day is believed to be able to modulate the expression of fibrosis genes and effectively inhibit AT1 receptors and prevent the formation of TGF-β1 to prevent type 1 collagen deposition, prevent fibrosis, and enhance the tissue regeneration process. ^22^ Administration of losartan as anti-fibrotic drugs can be used in most chronic inflammatory diseases, especially in heart and kidney disease. ^21^^–^^23^ The use of the ARB losartan to prevent fibrosis in acute conditions such as priapism can be a new finding and an attractive alternative in the management of daily practice.

Although the levels of TGF-1 on day ten and day 28 in the group receiving losartan were lower than the control, this was not statistically significant when compared to the control group (p day 10 = 0.3 and p day 28 = 0,17). Many factors may have an effect, one of which is the dose and duration of monitoring. If you look at the tendency for TGF-1 concentrations to continue to decrease on day 28 compared to day ten in the treatment group, the administration of losartan with a longer duration can reduce the level of TGF-β1 to a statistical significance.

The concentration of type 1 collagen also appeared to decrease with the administration of losartan on both the 10th and 28th days of observation. Then through calculations, it was proven that this was statistically significant between the group receiving losartan therapy when compared to the control group (p day 10 = 0.002 and p 28th day = 0.01). This research is in line with research by Varo *et al*, that administration of angiotensin inhibitors can inhibit type 1 collagen deposits and restore the process of tissue fibrosis. ^23^

There was no statistically significant difference in TGF beta levels in this study in line with research conducted by Frimpong *et al.* In his study, it was found that total circulating levels of TGF beta did not change with ARB administration but type 1 collagen levels decreased significantly. It is interesting to note in this study that the decrease in type 1 collagen levels is believed to occur due to a change in the ratio of active and inactive TGF beta of up to 90% which results in a decrease in type 1 collagen deposition and reduced fibrosis. ^10^

In addition, as we know the formation of type 1 collagen is a result of balancing various fibrotic and anti-fibrotic pathways. One of the cytokines that play a role in type 1 collagen deposition is the Tissue Inhibitor of Metalloproteinase-1 (TIMP-1). Meanwhile, the cytokine that causes the degradation of collagen is Matrix Metalloproteinase (MMP). MMP consists of collagenase, stromelysin, and gelatinase. TIMP-1 is generated by signals from inflammation, while MMP is produced by tissues constantly. TIMP-1 forms a balance with MMP in regulating type 1 collagen levels in tissues. If TIMP-1 is overexpressed, fibrosis will occur. Angiotensin II has a role in increasing TIMP-1 so that giving Losartan as an angiotensin inhibitor, can reduce TIMP-1 concentrations and restore MMP/TIMP-1 balance and able to reduce type 1 collagen deposition and prevent fibrosis by various pathways.

In this study, the approach used was the ability of losartan to suppress the action of profibrotic cytokines such as TGF-β1, but its ability to increase anti-fibrotic cytokines such as MMP which can degrade collagen has not been assessed. In addition, the effective dosage and duration of the use of losartan may need to be studied further to obtain maximum results. This can be a good suggestion for further research to determine the effectiveness of losartan in preventing fibrosis in priapism.

### Metavir score

Several studies have shown a reduction in hepatic fibrosis due to hepatitis C with losartan therapy. ^24^ In a retrospective human study, ACE-I/ARBs were able to reduce liver fibrosis in patients with chronic liver disease. In patients with hepatitis C virus infection who had undergone liver transplantation, the fibrosis stage and fibrosis progression index on liver biopsy were significantly lower in patients treated with ACE-I/ARB than in those not treated with ACE-I/ARB.

Histopathological examination is the gold standard for fibrosis and it can be assessed using fibrosis score. In one meta-analysis, there were two randomized controlled trials (RCTs) evaluating fibrosis scores. The results showed that ARB therapy had a trend of positive effects on liver fibrosis as assessed by the Metavir score. ^25^

ARB studies in cardiac fibrosis were performed using high doses of Telmisartan (5 mg/kg/day) and low doses (0.5 mg/kg/day) for five weeks. Myocardial fibrosis was significantly decreased in both groups (high dose and low dose). Perivascular fibrosis is also prevented by ARB. The use of ARBs does not decrease the serum angiotensin II concentration. However, aldosterone concentrations were significantly decreased. This suggests that aldosterone changes may be another influential factor in the prevention of cardiac fibrosis and hypertrophy. ^26^

Several experimental studies demonstrated the inhibitory effect of AngII on reducing skeletal muscle fibrosis. Losartan can reduce fibrosis and improve skeletal muscle strength in animal models of congenital muscular dystrophy and Marfan syndrome.

The role of AngII in fibrosis is seen in the presence of excessive induction of renin and AT. In addition, the synthesis of the extracellular matrix increases and AngII induces an increase in the concentration of collagen and fibronectin. Connective tissue growth factor (CTGF) appears as a profibrotic cytokine. CTGF is a mediator that stimulates TGF-β1 resulting in proliferation and activation of fibroblasts. ^27^^,^^28^

The fibrogenic effect of AngII is related to the activation of TGF-β1 signaling. Over-activation of TGF-β1 causes fibrosis in almost all tissues. TGF-β1 stimulates myofibroblast differentiation and extracellular matrix synthesis. TGF-β1 also acts to maintain extracellular matrix proteins by inhibiting the activity of matrix metalloproteinases (MMPs) and inducing the synthesis of tissue inhibitor metalloproteinases (TIMPs). ^29^

AngII inhibitory mechanism can block TGF-β1 signaling. Losartan anti-fibrotic effect occurs through the mechanism of suppression of TGF-β1 levels via the AngII type I receptor. Using a dose that had minimal effect on mean arterial blood pressure (MABP), it was shown that losartan reduced collagen I levels. ^30^

Another study also demonstrated a superior anti-fibrotic effect of ARBs compared to ACE-I on liver tissue fibrosis. ^31^ Long-term administration of ACE-I increases compensation. The accumulation of bradykinin due to ACE inhibition by ACE-I can lead to the development of fibrosis and activate TGF-β1, as well as synthesize extracellular matrix proteins. ^32^

However, findings related to the effect of ACE-I/ARB on fibrosis are still contradictory. Another study on liver fibrosis showed that losartan could increase the stage of liver fibrosis in chronic hepatitis C patients. Long-term use of ACE-I/ARBs for hepatitis C patients has not been shown to inhibit the development of liver fibrosis. ^25^^,^^33^

Contradictions have also been found in studies of the use of ARBs in interstitial lung disease. A study suggests a possible harmful role of renin-angiotensin inhibition in interstitial lung disease. The use of ARBs was associated with a 1.9-fold increase in the likelihood of having lung interstitial abnormalities at ten-year follow-up. It is suspected that ARB-induced compensation causes an increase in renin levels increasing angiotensin II. Renin itself increases the synthesis of collagen and TGF-β1 via an angiotensin II-independent mechanism. However, studies are still limited. ^34^

There are some limitations of the research. First, there was no comparison between the use of losartan and proven anti-fibrotic such as phosphodiesterase-5 inhibitors and pentoxifylline. It is necessary to compare with other anti-fibrotic agents to measure the effectiveness of losartan as an anti-fibrotic. Second, although using minimum sample calculation using federer formula, the sample still quite low. Last, further research is needed on the minimum dose required to provide anti-fibrotic effects in humans. The side effects of losartan anti-fibrotic doses in humans also need to be evaluated to prevent toxicity and unwanted side effects.

## Conclusion

Oral administration of the ARB losartan can reduce the concentration of TGF-β1 in male Wistar rats model priapism although this is not statistically significant and oral administration of the ARB losartan decreased the concentration of collagen type I statistically in male Wistar rats model priapism. Losartan administration also can prevent corpora cavernosa fibrosis in priapism model Wistar rats assessed by metavir score.

## Data availability

Mendeley Data. The Role of Angiotensin Receptor Blocker (Losartan) on Decreasing Fibrotic Process of Corpora Cavernosa in Priapism Model of Wistar Rats. DOI: https://doi.org/10.17632/7dpb82y8j5.1 ^35^

This project contains the following underlying data:

- Methods: A total of eighteen male Wistar rats mean were divided into 5 groups. For the priapism models, we applied negative pressure on the penis to make an artificial erection to mimic the priapism process. The control groups were observed and the treatment groups were orally given Losartan 15 mg/kg/day. Corpora cavernosa was harvested for TGF-β1 and collagen-type-1 measurement using an enzyme-linked immunosorbent assay (ELISA). The fibrotic tissue of each rat was then collected and assessed histopathologically with the Metavir scoring system

Data are available under the terms of the Creative Commons Attribution 4.0 International license (CC-BY 4.0).

## Reporting guidelines

Mendeley data. ARRIVE checklist. DOI: https://doi.org/10.17632/7dpb82y8j5.2

Data are available under the terms of the Creative Commons Attribution 4.0 International license (CC-BY 4.0).
