# Supplementary material for: The role of angiotensin receptor blocker (losartan) on decreasing fibrotic process of corpora cavernosa in priapism model of wistar rats
Source: F1000Res. 2024 Feb 6;11:831. Originally published 2022 Jul 26. [Version 3] doi: 10.12688/f1000research.123040.3 (PMC10690033; doi:10.12688/f1000research.123040.3)
Supplement: Peer review history

# Reviewer report: Reviewer response for version 2

**DOI:** 10.5256/f1000research.147597.r223719

**Published:** 2023-11-30

**Authors:** David J. Ralph

**Affiliations:** 1 Andrology Department, University College London Hospital, London, UK

The author present a manuscript that describes an antifibrotic effect of angiotensin receptor blockers in a rat model of priapism. They derive their conclusions from decreased levels of collagen I, which were established by ELISA, and the metavir score, which is typically reserved for assessment of hepatic fibrosis.

 There are numerous issues that need to be addressed/clarified.

- How was the drug dose selected? Is this dose achievable and safe in human subjects?
- Despite presenting this as an exploratory study, the n-numbers are quite low, which is a limitation that should be acknowledged.
- The manuscript needs minor text changes throughout to improve the quality of writing.
- The authors use both: antifibrotic and anti-fibrotic, please choose on and use throughout the entire manuscript.
- How was the losartan administered? Oral gavage or in drinking water? If drinking water, how was uptake/serum level confirmed?
- The ketamine dose seems very low at 90mg/kg for euthanasia, given that this is a safe dosage to anaesthetise pregnant rats (PMID:521380).
- In the TGF-beta1 and collagen assessment section in the methods: was 2,000 or 3,000rpm used? Why is there a range?
- The metavir scoring is not explained sufficiently. If this forms such a major part of their results and conclusions, then the methods need to reflect this and include a lot more detail on how the scoring was performed.
- Was the observer blinded during metavir scoring?
- TGF-beta1 shows no decrease after 28 days, it stays virtually the same as day 10 and is still above control levels. The authors presentation of this as a decrease is confusing.
- Tables 3&4 should also include the healthy control. The authors should also consider using ANOVA with multiple comparisons for statistical analysis here.
- Table 6 shows that there is no statistically significant difference between losartan and sham groups. Again, ANOVA with multiple comparisons might be more appropriate to analyse and interpret these results.
- The discussion says that proliferation and remodelling happen at days 10 and 28, respectively and make reference to ref 19. Reference 19 does not support these statements. 
- Further, are days 10 and 28 applicable to the human or rat situation?
- The discussion then mentions the Garg paper, which is ref 21, not ref 20. Please correct, as ref 20 does not support the statement made.
- Can the authors comment on the use of losartan in erectile dysfunction and how this fits with their data?
- The metavir score section in the discussion needs to be reworded. Specifically: "Histopathological examination of the liver is the gold standard for fibrosis". This might apply for hepatic fibrosis, but examination of the liver is not the gold standard for all fibrosis.

Is the work clearly and accurately presented and does it cite the current literature?

Partly

If applicable, is the statistical analysis and its interpretation appropriate?

Partly

Are all the source data underlying the results available to ensure full reproducibility?

Yes

Is the study design appropriate and is the work technically sound?

Partly

Are the conclusions drawn adequately supported by the results?

Partly

Are sufficient details of methods and analysis provided to allow replication by others?

Partly

Reviewer Expertise:

Andrology

I confirm that I have read this submission and believe that I have an appropriate level of expertise to confirm that it is of an acceptable scientific standard, however I have significant reservations, as outlined above.

## References

[1] : Ketamine/xylazine anesthesia in the pregnant rat. *J Am Vet Med Assoc* .1979;175(9) :952-3 521380

# Reviewer report: Reviewer response for version 1

**DOI:** 10.5256/f1000research.135103.r154482

**Published:** 2022-11-21

**Authors:** Guiting Lin

**Affiliations:** 1 Knuppe Molecular Urology Laboratory, Department of Urology, School of Medicine, University of California San Francisco, San Francisco, CA, USA

The manuscript in title of “The role of angiotensin receptor blocker (losartan) on decreasing fibrotic process of corpora cavernosa in priapism model of wistar rats” aims to explore the effect of losartan in reducing fibrosis in priapism by evaluating TGF-β1 and collagen-type-1 in cavernous tissue and determined the effect of losartan in preventing fibrosis in priapism model of Wistar rats assessed by the metavir score. This is a very interesting study. However, there are several issues need be revised extensively.

1. The sample size is too small, there is only a total 18 animals in 5 groups. Only 2 rats in normal control group. The current research findings are not reliable.
2. Please provide detail information about the groups, including the animal number in each group.
3. Please provide the full name of the abbreviation the first time it appears in the manuscript.
4. Please provide the statistical analysis information of the table, such as P value, N, etc.
5. Please provide images in high resolution >300 dpi.
6. Figure 3 should be re-organized, please provide a professional Figure.
7. Figure 4 is unqualified. Please compare the same area of penile erectile tissue and use the same magnification. Please add the scale bar. More importantly, please provide images for each group.

Professional English editing is recommended.

Is the work clearly and accurately presented and does it cite the current literature?

Yes

If applicable, is the statistical analysis and its interpretation appropriate?

No

Are all the source data underlying the results available to ensure full reproducibility?

Partly

Is the study design appropriate and is the work technically sound?

No

Are the conclusions drawn adequately supported by the results?

Partly

Are sufficient details of methods and analysis provided to allow replication by others?

No

Reviewer Expertise:

NA

I confirm that I have read this submission and believe that I have an appropriate level of expertise to confirm that it is of an acceptable scientific standard, however I have significant reservations, as outlined above.

# Response

**Published:** 2023-04-28

**Authors:** Dicky Stefanus

**Affiliations:** Padjadjaran University, Indonesia, Indonesia

Greetings, I am very sorry for the late response.

1. This study uses the Federer formula to decide the minimum sample size of each group (4 animals).
  In the normal control group (sham group) we only used 2 samples because we consider the animal welfare law from the Institutional Animal Care and Use Committee (IACUC) guidance.
2. Group 1: sample 1.1, 1.2, 1.3, 1.4 (a model of priapism without therapy which was sacrificed on day ten)Group 2: sample 2.1, 2.2, 2.3, 2.4 (a model of priapism without treatment which was sacrificed on day 28).
3. Group 3: sample 3.1, 3.2, 3.3, 3.4 (a model of priapism with the ARB losartan therapy sacrificed on day ten).
4. Group 4: sample 4.1, 4.2, 4.3, 4.4 (a model of priapism with the ARB losartan therapy sacrificed on day 28).
5. Group 5: sample 5.1, 5.2 (healthy controls).
6. Full name of the abbreviation in this manuscript has been added when it appears for the first time.
7. Tables 1 and 2 don’t need statistical analysis. while Table 3-6 already have their respective statistical analysis results.
8. Figures have been replaced with better resolutions images.
9. Figure 3 has been revised as per the instructions.
10. Figure 4 has been revised as per the instructions.

# Reviewer report: Reviewer response for version 1

**DOI:** 10.5256/f1000research.135103.r150826

**Published:** 2022-10-03

**Authors:** Widi Atmoko

**Affiliations:** 1 Department of Urology, Faculty of Medicine, Cipto Mangunkusumo General Hospital, University of Indonesia, Jakarta, Indonesia

Congratulations and well done to all the authors for finishing a well-written manuscript. The authors presented an experimental animal study on the effect of Losartan on TGF-β1 expression and collagen type-1 deposition due to low-flow priapism in rats. The topic chosen by the authors is novel and intriguing. The current approach to low-flow priapism has not considered using ARB to prevent fibrosis. Thus the finding of the study will provide novelty to the body of medical literature.

 The whole manuscript is clear and concise. The title and the abstract adequately represent the study's concept and finding. The objectives of the study are very clear and the methodology is also sound and reproducible. All the findings were correlated to the previous study. The figures are clear and legible.

 However, further research will still be needed to confirm its finding due to the small number of samples and the nature of the animal study. Nevertheless, this research will open a new horizon and possibility to a medical or non-surgical approach for low-flow priapism, at least for adjunctive treatment to prevent fibrosis due to priapism.

Is the work clearly and accurately presented and does it cite the current literature?

Yes

If applicable, is the statistical analysis and its interpretation appropriate?

Yes

Are all the source data underlying the results available to ensure full reproducibility?

Yes

Is the study design appropriate and is the work technically sound?

Yes

Are the conclusions drawn adequately supported by the results?

Yes

Are sufficient details of methods and analysis provided to allow replication by others?

Yes

Reviewer Expertise:

Andrology, male infertility, male sexual dysfunction, priapism, erectile dysfunction, ejaculatory and orgasmic problem, endoruology, urinary tract stone disease,

I confirm that I have read this submission and believe that I have an appropriate level of expertise to confirm that it is of an acceptable scientific standard.

# Response

**Published:** 2022-10-04

**Authors:** Dicky Stefanus

**Affiliations:** Padjadjaran University, Indonesia, Indonesia

Thank you for the review, yes we hope this research will make a pilot study with larger sample to other research in the future,

 Many thanks
